# The proteomic profile of whole and glandular saliva in healthy pain-free subjects

**DOI:** 10.1038/srep39073

**Published:** 2016-12-15

**Authors:** Hajer Jasim, Patrik Olausson, Britt Hedenberg-Magnusson, Malin Ernberg, Bijar Ghafouri

**Affiliations:** 1 Section of Orofacial Pain and Jaw Function, Department of Dental Medicine, Karolinska Institutet, and Scandinavian Center for Orofacial Neurosciences (SCON), SE 14104 Huddinge, Sweden; 2 Folktandvården Stockholm AB, Sweden; 3 Division of Community Medicine, Department of Medical and Health Sciences, Faculty of Health Sciences, Linköping University and Pain and Rehabilitation Center, Anaesthetics, Operations and Specialty Surgery Center, Region Östergötland

## Abstract

Determination of the variability in the salivary proteome is a prerequisite for the development of saliva as a diagnostic and prognostic tool in particular physiological states. In this context, it is important that technical variability induced by sample collection and processing is kept at minimum to be able to reproducibly assess variability in states of health and disease. In the current study, the proteome profile in unstimulated and stimulated whole, parotid and sublingual saliva was investigated using two-dimensional gel electrophoresis. Saliva samples were structurally collected from ten examined and characterized healthy individuals during the exactly same conditions. The results demonstrated that different collection methods provide clear differences in the snapshot of the salivary proteome and also in the relative amount of specific proteins. The variable nature of the salivary proteome suggests that different approaches may have to be adopted when studying its composition or its possible role as an indicator for particular physiological states. The results emphasize the importance of consistency when collecting saliva samples for proteomic analysis.

Human saliva contains numerous proteins and peptides, each of them carries several significant biological functions. These proteins are not only important in maintaining the health of the oral cavity but also may yield information about both local and systemic disease^1^.

Functions of the saliva are not only restricted to processing food for digestion. Saliva contains a large number of proteins, which play important roles in the regulation of the immune defence, endocrine system and maintenance of mucosal tissue and dental health^2^,^3^. The salivary glands are also integrated into the neuroendocrine system through complex regulatory pathways^4^. Some of the peptides found in saliva are also present in spinal fluid and microdialysate, where they have been implicated in a proposed neurochemical pathogenesis of pain in rheumatoid arthritis^5^, temporomandibular disorders (TMD)^6^,^7^, trapezius myalgia and fibromyalgia syndrome^8^,^9^.

Ultimately, saliva may contain locally expressed proteins and other substances that can be used as indicators of diseases^4^,^10^. These components, called biomarkers, can be closely related to an individual’s health condition and can change greatly when diseases afflict. Using saliva for biomarker detection and prognosis is not only practical and non-invasive, but it could also provide a more accurate fluid than blood^10^. Unlike blood it does not clot and is easier to handle, also saliva contains a smaller quantity of proteins, therefore decreasing any potential risk of non-specific interference and hydrostatic interactions. Within blood, the protein concentration can vary over several orders of magnitude, with protein half-life’s ranging from a few seconds to several months or longer. The protein concentration in saliva is approximately a quarter of what is presented in blood which makes it easier to select and investigate low abundant proteins^11^,^12^. The composition of saliva is though not as complex or varying as serum, it should therefore more accurately reflect the current condition of the body at any given time. The advantages with saliva might facilitate early detection of many diseases, anticipate prognosis and improve clinical management^13^,^14^.

Although the proteins in saliva reflects the body’s health and well-being, its use as a diagnostic fluid has been hindered, mainly because of the lack of standardized techniques to collect saliva and comprehensive detection methods to study the protein content. Most studies using saliva as a diagnostic medium use different collection methods and often lack to define characterization of the patients or sampling procedure^4^,^15^,^16^. This make it difficult to compare results from different studies^17^.

In general, most studies view saliva wrongly as a homogeneous body fluid. However, saliva is not a solitary fluid and cannot be viewed as such. Rather, it is a complex mixture that comprises the secretions of three major glands (the parotid, submandibular and sublingual glands) each secreting a characteristic type of saliva, hundreds of minor salivary glands, gingival crevicular fluid and debris. It is also not stable but constantly in change and the composition is affected among other things by sampling methodology, environment, periodicity, oral hygiene, psychological status and general health^18^. As a diagnostic medium, saliva has disadvantages that needs consideration. For instance, owing to the diurnal variations of certain biomolecules present in saliva, it does not always reliably reflect the concentrations of these molecules in serum. Consequently, since many factors can influence on salivary secretion and composition a precise standard for saliva collection must be established^19^.

The aim of the current study was to in depth analyse and compare different saliva collection methods on a protein level using two dimensional gel (2-DE) based proteomic approach in a strictly controlled and characterized healthy cohort. To the best of our knowledge, this is the first study that structurally has analysed the salivary proteome in whole and glandular saliva.

## Materials and Methods

### Participants

Ten healthy participants, five men and five age-matched women, with a mean (SD) age of 23.6 ± 2.2 years where included in this study.

Inclusion criteria were good general health, age ≥18 years, not taking any medications including oral contraceptives, body mass index (BMI) < 30 kg/m^2^ and no complaints of oral dryness, mucosal lesions or other oral complaints. Participants had also to be free of fever/or cold and maintain exceptional oral hygiene on the day of collection.

Participants with less than 22 teeth and extensive prosthodontics rehabilitations were excluded. If oral examination indicated poor oral hygiene, hyposalivation, oral complaints (e.g. peritonitis, caries, tooth ache), mucosal lesions, clinical signs of ongoing periodontal diseases or dental attrition, they were directly excluded from further involvement in the study. Participants reporting elevated levels of psychological distress were also excluded.

Participants were requested not to eat, drink or brush their teeth 1 h prior to the trial, and not consume alcoholic beverages 24 h prior to collection. The participants arrived to the clinic in the early morning after they had eaten breakfast. A brief interview was carried out by the examiner to ensure that they had followed the instructions, which all had. Participants were then asked to fill in validated questionnaires and then a clinical examination was carried out as described below.

All participants received information regarding the objectives and procedures of the study and gave their informed written consent before the start of the study. The study protocol was approved by the Regional Ethical Review Board in Stockholm, Sweden (2014/17–31/3) and followed the guidelines according to the Declaration of Helsinki.

### Questionnaires and clinical examination

All participants underwent a dental examination by the same examiner. During the clinical examination participants were checked for attrition as a sign of bruxism, decayed teeth and periodontal diseases, as well as the occlusion. Participants were also evaluated by the Swedish version of the Diagnostic Criteria for TMD (DC/TMD) axis I and II^20^. The evidence-based protocol was used as a screening instrument for identification of participants with TMD signs that may not be presented during the interview and general examination. Participants showing clinical signs of TMD (except for disc displacement without reduction which was considered as normal fluctuation) were excluded from further involvement in the study.

The following brief screening instruments included in the DC/TMD axis II questionnaire were used to assess symptoms of depression, somatic symptoms, anxiety, mental stress and jaw function: the Patient Health Questionnaire (PHQ-9 and PHQ-15), the Generalized Anxiety Disorder scale (GAD-7), the Perceived Stress Scale-10 (PSS-10) and Jaw Functional Limitation Scale (JFLS).

The PHQ is a self-administered version of the Primary Care Evaluation of Mental Disorders diagnostic instrument for common mental disorders. PHQ-9 is a well validated instrument to estimate and determine the severity of depression^21^. The PHQ-9 evaluates nine symptoms of depression, and assesses the level of the depression by the frequency of these symptoms within the last 2 weeks. Scores range between 0 and 27, and scores of 5, 10 and 20 are considered the boundaries for mild, moderate, and severe depression respectively. The PHQ-15 is a somatic symptom subscale and inquiries about the frequency of 15 somatic symptoms or symptom clusters that account for more than 90% of the physical complaints (excluding upper respiratory tract symptoms). In determining the PHQ-15 score, each individual symptom is coded as 0, 1, or 2, and the total score ranges from 0 to 30^22^.

The GAD-7 was used to estimate and determine the severity of anxiety. The GAD-7 has been shown to be a reliable and valid measurement of anxiety and it can determine how often the participant has suffered from seven problems over the past weeks, including nervousness and worry, where the severity is evaluated by the total score of the questionnaire (range: 0–21; higher scores indicate more severe anxiety). Scores of 5, 10, and 15 are considered the cutoffs for mild, moderate, and severe anxiety^23^.

The PSS-10 is a well document instrument to tap how unpredictable, uncontrollable and overloaded participants find their lives. Participants rate the items on a 5-point Likert scale (0–4) where higher scores reflect greater perception of stress^24^. The PSS final score is obtained by reversing the scores on the five positive items, and then summing all 10 items. The total score ranges from 0 to 40. Higher scores indicate higher perception of stress.

The JFLS is designed to assess patients jaw functional level that is both joint-specific and separated from pain-related disability. It has 20 items that address mastication, jaw mobility, and verbal and emotional expression. Each item is rated on a numeric rating scale of 0 to 10 (where 0 indicated no limitation and 10 indicated severe limitation)^25^.

### Saliva collection

**Figure 1:**
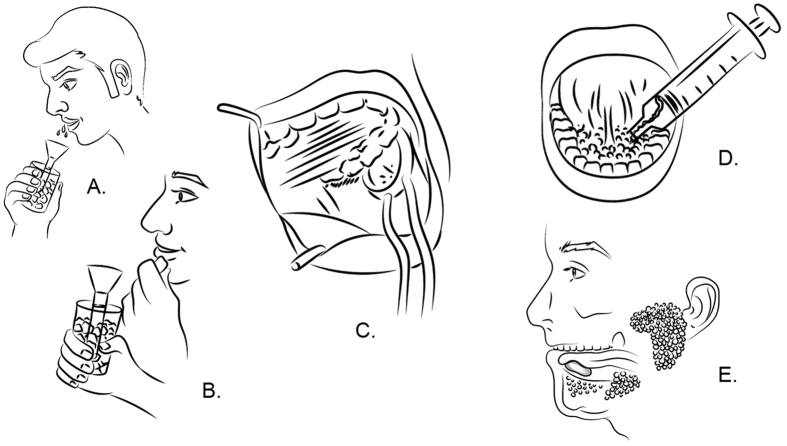
Illustrative overview of the different saliva salivary collection approaches compared in the study. (**A**) Unstimulated whole saliva, (**B**) Stimulated whole saliva, (**C**) Unstimulated and stimulated parotid saliva, (**D**) Unstimulated sublingual saliva, (**E**) Stimulated sublingual saliva.

Unstimulated and stimulated whole, parotid, and sublingual saliva was sampled (Fig. 1). Before saliva collection, participants were instructed to rinse their mouth with 10 ml of distilled deionized water for 30 seconds to remove debris and moisturise the mucosa. After 10 minutes of rest, saliva collection started. Between every sampling, participants rested for 15 minutes to neutralize salivary flow. During the collection, participants were asked to not speak or leave the room. Further, they were instructed to keep their eyes open during salivation and not actively stimulate salivary flow.

All saliva samples were collected in the same order as presented below, in the same clinical room and at the same time, between 8:30 and 10:30 am. To prevent degradation of sensitive proteins all samples were collected on ice in precooled polypropylene tubes. Immediately after collection a Protease Inhibitor Cocktail (Sigma Aldrich v/v 1:500) was added. All samples where then centrifuged at 700 × g for 15 minutes at 4 °C to remove debris. The supernatant of each sample (upper 2/3) was fractionated into 100 μl tubes and frozen at −70 °C until analyses.

#### Unstimulated whole saliva

Participants were instructed to sit upright with their head slightly titled forward. A 5 ml polypropylene tube was used to collect up to 5 ml of saliva during passive drooling. Total drooling time was recorded, and salivary flow was measured.

#### Unstimulated parotid saliva

The parotid gland secretion is voided in the oral cavity via the Stensen’s duct at the vicinity of the parotid papilla opposite the maxillary second molar. In order to collect pure parotid saliva a modified polymethylmethacrylate Carlsson-Critten collector was used according to authors’ description^26^,^27^. The orifices of the parotid duct was located, and the surrounding buccal mucosa dried with a gauze. The collector was placed bilaterally on the buccal mucosa surrounding Stensen’s duct and secured with suction using a syringe. Parotid saliva were collected via 25 cm plastic tubing into a precooled tube. The first 3 drops of saliva were discarded to reduce the possibility of contamination. Total drooling time was recorded, and salivary flow was measured.

#### Unstimulated sublingual saliva

The submandibular and sublingual gland secretions are voided in the oral cavity mainly via the Wharton duct and several smaller sublingual ducts which opens into the floor of the mouth.

By blocking the Stensen’s duct using Carlsson-Critten collector as described above, saliva could be collected from the floor of the mouth with a syringe every second minute. Samples from the first 2 minutes were discarded to neutralize salivary flow.

#### Stimulated parotid saliva

The Carlsson-Critten collector was placed bilaterally over the Stensen’s ducts orifices. Sterile aqueous 2% citric acid solution was applied bilateral on the lateral aspects of the tongue, at intervals of 30 seconds using a sterile cotton swab (max total 300 μl). Up to 1 ml parotid saliva was collected as described above (unstimulated parotid saliva).

#### Stimulated sublingual saliva

Saliva Bio Oral Swab^®^ (Salimetrics) consist of a polypropylene tube and contains absorbent pad made by synthetic material. The pad was placed for around 2 minutes under the tongue while stimulating with 2% Citric acid as described above until the swab was fully covered in saliva. The fluid was obtained by centrifugation (1500 × g, 15 min, 4 °C). Up to 2 ml saliva was extracted from the swab.

#### Stimulated whole saliva

Prior to collecting the last sample, participants were instructed to again rinse their mouth with 10 ml of distilled deionized water for 30 seconds to remove any remains of the citric acid. Saliva samples were collected using paraffin gum (Orion Diagnostica, Finland). For prestimulation, the participants were instructed to chew the gum until it was smooth and flexible. After 60 seconds of prestimulation, the participants were asked to swallow the saliva present in the mouth. Subsequently, around 5 ml of whole saliva, stimulated by the same piece of paraffin, was collected for around 2 minutes, and salivary flow rate was measured.

### Protein analyses

The protein concentration was determined by two dimensional gel electrophoresis (2DE) using the Bio-Rad protein assay according to Bradford (Bio-Rad, Hercules, CA, USA). Aliquots (300–400 μl) of the samples were desalted by gel filtration (PD-10 column, GE Healthcare) into 12 mM ammonium bicarbonate, pH 7.1. Proteins were lyophilized and dissolved in 0.20 ml urea sample solution according to Görg *et al*.^28^. 2-DE was performed in a horizontal 2-DE setup (IPGphore and Multiphore, GE Healhcare) as described in detail previously^29^. The samples (containing 50 μg protein) were applied by in-gel rehydration according to the manufacturer’s instructions for 12 h using low voltage (30 V) in pH 3–10 NL 18 cm IPGs (GE Heathcare, Sweden). The proteins were then focused for up to 32 000 Vhs at a maximum voltage of 8000 V to assure a steady state. IPGs were immediately stored at −70 °C until analysed.

The IPG gel strips were equilibrated in SDS equilibration buffer (urea 6 M, SDS 4% (w/v), glycerol 30.5% (w/v) and Trizma-HCl 50 mM) and DTT 1% (w/v) for 15 minutes and then with iodacetamide 4.5% (w/v) for additional 15 minutes. The second dimension (SDS-PAGE) was carried out by transferring the proteins to gradient gels (ExcelGel XL 245 × 180 × 0.5 mm, 12–14%T, 3%C) running at 20–40 mA, up to 1000 V for about 5 h.

### Staining and image analysis

For protein detection, the gels were stained with silver according to Shevchenko *et al*.^30^ with a detection limit of 5 ng/spot^31^ using a Stainer Shaker (Hoefer Processor Plus, Amersham Bioscience). The 2-DE protein patterns of the gels were visualized as digitized images using a CCD camera system, Versa Doc (Bio-Rad Hercules, CA, USA) in combination with a computerized imaging 12-bit system designed for evaluation of 2-DE patterns (PDQuest V 8.0.1; Bio-Rad). The amount of protein in a spot was assessed as background corrected optical density, integrated over all pixels in the spot and expressed as integrated optical density (IOD). In order to correct for differences in total silver stain intensity between different 2-DE images, the amounts of the compared protein spots were quantified as optical density for individual spot per total protein intensity of all spots in the same gel. Thus ppm-values (parts per million) for all proteins were generated, and were statistically evaluated for differences between collection methods.

The protein spots were identified by comparison to previously identified saliva proteins from a local database available at the laboratory^29^.

### Statistics

Differences between males and females in the study were tested with Mann-Whitney U-test using Statistica (StatSoft, Oklahoma, USA) since most of these variables did not show normal distribution. Repeated measurement analysis of variance (ANOVA) was used to analyses differences in saliva collection with Bonferroni test as post hoc test when the ANOVA indicated significant differences.

Correlations between variables were tested for statistical significance with Spearman correlation test, adjusted for multiple comparisons according to Bonferroni. Descriptive data are presented as mean and standard deviation (SD) or median and interquartile range (IQR). For all analyses, the significance level was set at P < 0.05.

When investigating the multivariate correlations between the proteins and group membership. Orthogonal Partial least squares discriminant analysis (OPLS-DA) was applied using SIMCA-P+ v.13.0 (UMETRICS, Umeå, Sweden)^8^,^32^,^33^. Principal component analysis (PCA), also available in SIMCA-P+ was used to identify multivariate outliers. No multivariate outliers were identified. The variable influence on projection (VIP) indicates the relevance of each X-variable pooled over all dimensions and the Y-variables – the group of variables that best explains Y. VIP > 1.2 was considered significant.

## Results

### Data overview

Anthropometric data of participants in the study are presented in Table 1. Small differences in body mass and number of erupted teeth were observed between males and females in the cohort. Females in the cohort reported overall higher levels of anxiety and somatic symptoms compared to the male equivalent (p = 0.032), however all psychological and psychosocial parameters were normal according to standard values. Otherwise there were no statically significant differences regarding demographical, psychological and salivary parameters between males and females (Mann-Whitney U-test).

Salivary flow differed significantly between collection methods (F = 68.852; P = 0.000; Repeated measurement ANOVA) and was not affected by gender (F = 1.304; P = 0.305; Repeated measurement ANOVA). As expected, whole paraffin stimulated saliva showed higher flow rate (2.2 ± 1.2 ml/min) compared to other collection methods (Repeated measurement ANOVA with Bonferroni post hoc test; P < 0.0001) (Table 2).

The intra and inter subject coefficient of variation (CV) was calculated to show the extent of variability in relation to the mean value in the protein concentration. For all six collection approaches the CV varied from 35% to 92%. Lowest CV was observed for stimulated whole saliva and the highest CV was observed for unstimulated whole saliva. The protein concentration between individuals displayed CV between 28% up to 86% and reflects the intra-subject variability that can be expected in cross sectional studies.

Contrary, no significant differences in protein concentration was found between unstimulated and stimulated salivary collection approaches (F = 1.899; P = 1.304: Repeated Measures ANOVA). The protein concentration ranged from 776 to 12 906 μg/ml (Table 2). There was no correlation observed between the protein concentrations of the different collection methods before and after desalting (P > 0.05; Spearman Rank Order Correlations).

Unstimulated parotid saliva displayed as expected very slow flow rate (0.029 ± 0.026 ml/min per gland). Due to the overall slow flow rate and great variations in salivary flow rate of parotid saliva, sufficient amount for analysis could not be collected from all participants. Thus, saliva was collected from accessible gland from seven subjects but sufficient volume for 2DE analysis could only be obtained from three subjects.

### Proteome patterns in the different saliva collection methods

**Figure 2:**
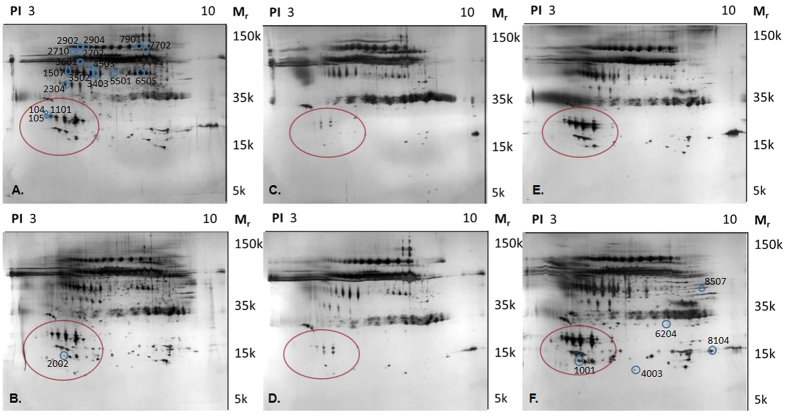
Representative protein patterns of different saliva collection methods. (**A**) Unstimulated whole saliva, (**B**) Stimulated whole saliva, (**C**) Unstimulated parotid saliva, (**D**) Stimulated parotid saliva, (**E**) Unstimulated sublingual saliva, (**F**) Stimulated sublingual saliva. The proteins were separated by two-dimensional gel electrophoresis (2-DE) and stained with silver with a detection limit of 5 ng/spot. The 2-DE protein patterns of the gels were visualized as digitized images using a CCD camera system in combination with a computerized imaging 12-bit system designed for evaluation of 2-DE patterns. The figures are scanned gels and are presented as un-manipulated images. Numbers refer to proteins in Table 3 and Fig. 4. The marked area represents proteins in pI and molecular weight range of 3–5 and 10–20 kDa respectively. This marked area illustrate the clearest differences between the different saliva samples.

The 2-DE protein patterns were visualized by silver staining and using spot detection wizards, 94 to 462 protein spots could be detected in each gel. Least number of protein spots were detected in the unstimulated and stimulated parotid saliva. The typical protein patterns for the different saliva samples are shown in Fig. 2. The protein pattern showed great differences between different sampling methods. The largest differences were detected in the *pI* range of 3 to 5 and molecular weight 10 to 20 kDa as marked by a circle in Fig. 2. There were significant lower levels of Prolactin-inducible protein and Cystatin S in unstimulated and stimulated parotid saliva compared to the other saliva samples.

**Figure 3:**
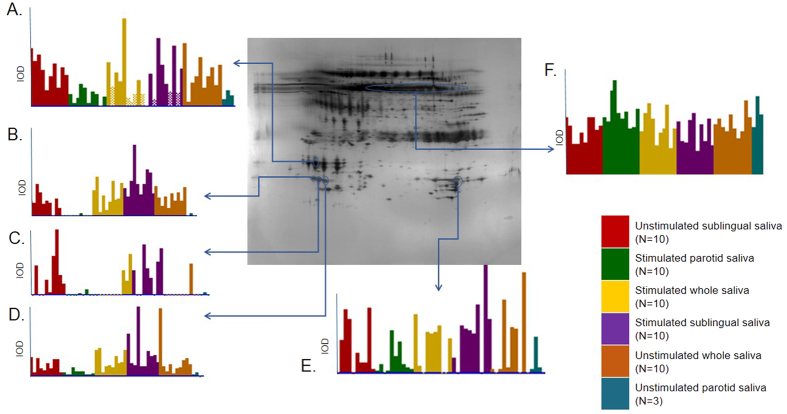
A typical two-dimensional electrophoresis (2-DE) gel map illustrating the salivary protein pattern and expression. The diagrams show the integrated optical density (IOD) of the protein in the individual gel from the different saliva samples. The different saliva samples are marked with different colors as indicated in the legend. N represents the number of subjects in each group. The diagrams illustrate the variation in the expression level of typical saliva proteins in the different saliva samples. (**A**) Prolactin-inducible protein, (**B**–**D**) isoforms of Cystatin S, (**E**) Cystatin SN), (**F**) Alpha-amylase.

A typical 2DE-map illustrating the salivary protein pattern and expression is shown in Fig. 3. The integrated optical density (IOD) of the typical salivary proteins (alpha-amylase, Cystatin S, Cystatin N and Prolactin-inducible protein) in the individual gel from the different saliva samples are shown in the diagrams. There were no differences in the level of alpha-amylase between the different saliva samples. A great individual variation was found for Cystatin SN in all saliva samples. The level of Prolactin-inducible protein and 3 different isoforms of Cystatin S were differentially expressed in the different saliva samples.

**Figure 4:**
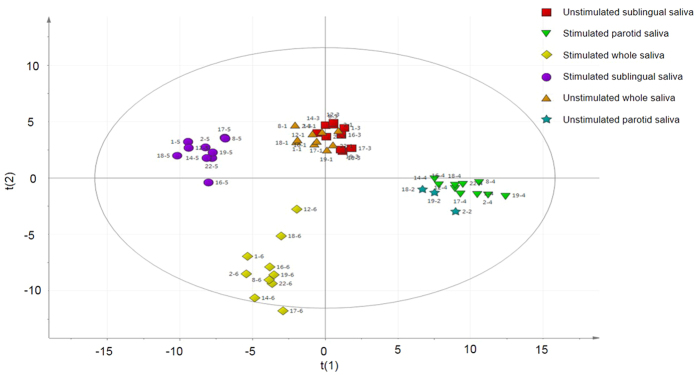
Orthogonal Partial least squares discriminant analysis (OPLS-DA) score plot showing a clear separation of the different saliva collection methods. The separations are based on the proteins tabulated in Table 3. The protein patterns of unstimulated sublingual saliva (red squares) and unstimulated whole saliva (orange triangles) are clustering together. Stimulated parotid saliva (green triangles) and unstimulated parotid saliva (green stars) are clustering together. The protein patterns of stimulated whole saliva (yellow squares) and stimulated sublingual saliva (purple circles) differs clearly from the other collection methods. Estimate of prediction Q2 = 0.79 and R2 = 0.93. The ellipse shows the 95% confidence interval using Hotelling T^2^ statistics.

A total of 213 protein spots were matched. At least 50% of these were presented in each salivary sampling methods and thus was eligible for statistical validation. 25 protein spots with VIP > 1.2 combined with jack-knifed 95% confidence intervals in the regression coefficients plot not including zero were considered significant (Table 3) and discriminated the different saliva collection methods (Fig. 4). Most spots of the 25 significant proteins were found to be identical to those identified in unstimulated whole saliva (Fig. 3A). Four spots (4003, 6204, 8104 and 8507) were more abundant in stimulated sublingual saliva. Spot number 1001 was found to be highly specific to stimulated whole saliva and stimulated sublingual saliva. Spot number 2002 was detected in almost all gels (n = 9) in stimulated whole saliva samples and only in 4 gels from unstimulated whole saliva and stimulated sublingual saliva and in 3 gels from unstimulated sublingual saliva. Unstimulated and stimulated parotid saliva gels lack the spot 2002.

## Discussion

Determination of the variability in the salivary proteome is a prerequisite for the development of saliva as a diagnostic and prognostic tool for biomarker discovery. In this context, it is important that technical variability induced by sample collection and processing is kept at minimum to be able to reproducibly assess inter-subject variability in states of health and disease^15^.

In the current study, the effect of unstimulated and stimulated saliva collection methods on the proteome profile was investigated. Using a small cohort and multiple samples allowed to detect major changes and reduced the influence of individual differences and external factors^34^. The participants were sex matched and closely related in age in order to minimize the influence of these factors on the flow rate and protein expression, since it has been described in earlier studies^35^. Salivary flow showed great differences in accordance with previous reported levels^36^,^37^,^38^ and was directly associated with the method for saliva collection.

The relative contributions of the different glands to whole saliva vary depending upon the method of collection, the degree of stimulation, age and even the time of the day^39^. The variable nature of saliva secretion suggest that different approaches may have to be adopted when studying its composition or its possible role as an indicator for particular physiological states. There is now a considerable body of literature on the diagnostic possibilities of saliva, but there are still no standardized techniques for saliva sample collection. In different studies, different sampling methods are often used and many articles provide no or little description about the preparation of the patient or sampling procedure^10^. Additionally, the characterization of the participants are often insufficient without a proper clinical examination. Most papers in saliva proteomics focus exclusively on studying whole saliva^1^,^15^,^29^ because it can be obtained very easily by spitting into a test tube or allowing it to dribble from the mouth. Very few have focused on ductal saliva obtained from the different salivary glands^16^,^17^,^40^. And, as far as we know, this is the first study focusing on structurally comparing the protein expression of both whole and glandular saliva in a carefully characterized and clinically examined cohort. The results demonstrated that different collection methods provided clear differences in the snapshot of the salivary proteome. Waltz and co-authors described differences in the protein expression between whole, parotid and sublingual-submandibular saliva^17^. Using a similar approach, but a much smaller cohort (n = 4), they reported less protein spots in glandular saliva compared to the present study. Though, the authors chose to filter saliva prior to desalting with the purpose of removing large insoluble material and bacterial contamination. This could consequently lead to loss of proteins and should not be needed if the participants are examined clinically for exclusion of oral disease and neglected oral hygiene prior to collection^17^. In another more recent study, also in a small uncharacterized cohort (n = 6), significantly different proteoform profiles were resolved with high reproducibility between stimulated parotid and submandibular/sublingual secretion^16^.

As indicated in earlier studies, samples collected by the same method displayed closer correlation than those obtained from different methods^36^. Our results revealed close correlation between the protein patterns of stimulated parotid saliva and unstimulated parotid saliva as well as unstimulated sublingual saliva and unstimulated whole saliva. The parotid gland’s main function is carbohydrate digestion and formation of the food bolus, consequently serous cells predominate, making the gland secrete mainly serous secretory product high in salivary alpha-amylase and electrolyte content. This may explain the somehow low level of specific protein spots and high level of salivary alpha-amylase in parotid saliva. The main functions of sublingual saliva are to lubricate the oral cavity and protect against chemical and mechanical influence. The saliva is dominated by mucous cell secretion which makes it rich in mucins and other glycoproteins. The glycoproteins (e.g. mucins) are high molecular weight polypeptides that stick together and have very low solubility, and cannot migrate through the polyacrylamide gel resulting in their aggregation^41^,^42^. This may in turn contribute to the somehow lower level of specific protein spots.

The close relationship between unstimulated sublingual saliva and unstimulated whole saliva may be due to the high content of sublingual saliva in the unstimulated whole saliva. The major component of unstimulated saliva is derived from the submandibular glands (60%), followed by the parotid glands (20%), minor salivary glands (15%), and sublingual glands (5%)^43^. The submandibular glands are responsible for producing high concentration of glycoproteins which consequently makes unstimulated whole saliva and unstimulated sublingual saliva viscous and difficult to technically process.

The protein pattern of stimulated whole saliva and stimulated sublingual saliva, however, differed significantly. This finding may be explained by the great differences in salivary quality and stimulation method. Stimulated sublingual saliva was collected with an absorbent material under the tongue while chemically stimulating with 2% citric acid on the lateral aspect of the tongue, while stimulated whole saliva was collected through mechanical stimulation by chewing on paraffin gum. The parotid gland normally responds to stimulation and proprioceptive activity, via masticatory muscles and periodontal ligaments, and accordingly contribute mostly to the stimulated whole saliva (60%)^37^,^41^. In addition to the differences in the proteomic profile, the relative amount of expressed proteins differed between salivary methods. The strength of electrophoreses based proteomics is that it allow us to separate and study different protein isoforms^34^. The finding that the same protein, e.g. Cystatin S and Prolactin-inducible protein, was identified in different spots in different saliva types suggests the presence of several isoforms. The amount of each isoforms also showed different expression in different saliva collection approaches. A typical example is the spot number 104 with the highest VIP-value (Table 3) that was highly present in unstimulated whole saliva and sublingual saliva but not in parotid saliva. The next spot number with high VIP-value, 105, was highly expressed only in unstimulated whole saliva and stimulated sublingual saliva. Both protein spots were identified as Prolactin-inducible protein. This protein is a common saliva protein that is secreted as glycosylated and non-glycosylated protein and has a roll in innate immunity and mucosal defence^44^,^45^. Accordingly, when using saliva as diagnostic or prognostic fluid, scientists need to carefully consider the saliva collection approach. This is highly important when studying the salivary proteome profile for particular physiological states in order to find biological markers.

Although superficially it may appear that the collection of saliva is a simple non-invasive procedure, there are limitations with the different collection methods that may not be obvious. The parotid glands are known to secrete several organic and inorganic substances. The saliva composition is highly viscous and easy to process. The collection is however somehow time-consuming and require special devices e.g. parotid cups^26^. Unstimulated parotid saliva presents very low flow-rate and is consequently not suitable when larger sample volumes are needed. This collection method is thus not practical in large cross-sectional studies or clinical studies involving patients with serious health conditions. The submandibular and sublingual glands are closely situated and can sometimes share the same ducts. Therefore, it is difficult to separate the saliva from these glands untraumatically and with certainty. This is the reason why saliva were sampled from both glands together in the current study^46^. The use of absorbent materials to collect saliva is common in saliva research but could be problematic when the expected volume of saliva is small, and can negatively affect the validity of the assays when the saliva is filtered through a cotton/pad. The collection of unstimulated whole saliva, by simply allowing saliva to accumulate in the mouth and then allowing it to dribble out through a funnel or straw in a tube, is subject to considerable variation as the participants tries, to varying extents, speed up the collection by tongue and cheek movements. This could partly explain the large variability in saliva secretion and protein concentration between subjects. In order to diminish that risk it is recommended that researchers give clear instructions to which participants must adhere before and during saliva collection. Paraffin-stimulated whole saliva could therefore be recommended due to its simplicity. This collection method also resulted in the least variability (CV 35%), the highest volume of saliva in this study and low levels of glycoproteins. In spite of these limitations, there are compelling reasons for exploring saliva as a diagnostic tool in proteomics to identify specific protein biomarkers for diseases.

In conclusion the results of this study demonstrated that different saliva collection methods provide clear differences in the snapshot of the salivary proteome. Based on the comparison of unstimulated and stimulated salivary collection approaches, it could be suggested that stimulated whole saliva may be a preferable collection method based on the simplicity of the collection method and low variability. The protein expression showed thus great variability between methods. The results emphases the importance of consistency when collecting saliva samples, which should be more important than the collection approach itself.

## Additional Information

**How to cite this article**: Jasim, H. *et al*. The proteomic profile of whole and glandular saliva in healthy pain-free subjects. *Sci. Rep.* **6**, 39073; doi: 10.1038/srep39073 (2016).

**Publisher's note:** Springer Nature remains neutral with regard to jurisdictional claims in published maps and institutional affiliations.

## Figures and Tables

**Table 1:** Overview of the participants in the study (n = 10).

Variable	Males (n = 5)	Females (n = 5)	P-value
Age (Years)	23.7 ± 2.3	23.5 ± 2.1	0.841
Body Mass Index (kg/m^2^)	24.0 ± 3.8	21.9 ± 3.1	0.301
Number of teeth	28.6 ± 0.9	30.8 ± 1.8	0.096
PHQ-9 Score	0 (0)	1 (0)	0.222
PHQ-15 Score	1 (1)	3 (1)	0.032
GAD-7 Score	0 (0)	1 (1)	0.032
PSS-10 Score	5 (3)	9 (4)	0.151
JFLS Score	0 (0)	0 (0)	0.690

Questionnaire scores are described as median (IQR) unless other is stated.

Aberrations; N = number of subjects in each group; PHQ = The Patient Health Questionnaire: GAD = Generalized Anxiety Disorder; PSS = Perceived Stress Scale; JFLS = Jaw Functional Limitation Scale.

**Table 2:** Description of mean (±SD) salivary flow, protein concentration and number of detected protein spots in different salivary collection approached among participants (n = 10).

	Parotid saliva*		Sublingual saliva		Whole saliva	
	Unstimulated	Stimulated	Unstimulated	Stimulated	Unstimulated	Stimulated
SALIVARY FLOW (ML/MIN)	0.029 ± 0.026	0.222 ± 0.284	0.14 ± 0.063	MD	0.18 ± 0.075	2.18 ± 1.164
PROTEIN CONCENTRATION (μG/ML)	4503 ± 4135	2837 ± 1416	1999 ± 823	1797 ± 847	2479 ± 1537	2171 ± 764
NUMBER OF PROTEIN SPOTS	164 ± 37	125 ± 26	304 ± 84	230 ± 59	155 ± 10	259 ± 84

MD = missing data.

*Salivary flow is reported unilaterally.

**Table 3:** Discriminant proteins between the different saliva collection methods in healthy participants.

SPOT NO	VIP	Parotid saliva		Sublingual saliva		Whole saliva	
		Unstimulated	Stimulated	Unstimulated	Stimulated	Unstimulated	Stimulated
0104 **PROLACTIN INDUCIBLE PROTEIN**	1.71	—	—	2988.0 (2126.44) N = 10	2434.09 (2202, 96) N = 10	1220.01 (838.06) N = 9	287.15 (75.87) N = 2
0105 **PROLACTIN INDUCIBLE PROTEIN**	1.65	—	—	273.20 N = 1	3253.50 (493.76) N = 4	2594.71 (871.97) N = 8	—
7901 **IG A**	1.59	2482.8 (2058.95) N = 2	1188.8 (237.72) N = 3	3215.11 (4007.45) N = 9	1745.21 (1410.92) N = 7	2168.50 (2590.51) N = 9	1603.02 (1198.34) N = 10
3502 **ZN-Α-GLYCOPROTEIN**	1.49	7963.53 (2409.24) N = 3	4234.48 (1776.09) N = 10	6895.01 (3871.87) N = 10	6877.32 (3883.61) N = 9	4101.38 (2040.10) N = 10	5669.56 (2412.18) N = 9
8104 **CYSTATIN SN**	1.43	—	268.125 (481.49) N = 8	910.52 (614.10) N = 7	1048.4 (418.59) N = 8	1638.12 (1398.74) N = 9	2032.14 (4084.58) N = 10
**ZN-Α-GLYCOPROTEIN**	1.38	—	1431, 00 (901.98) N = 3	744.35 (475.45) N = 4	515.32 (183.11) N = 5	811.64 (614.19) N = 7	389.62 (367.96) N = 5
2304 **SPLUNC 2**	1.36	229.85 (76.29) N = 2	—	195.35 (63.7) N = 2	159.328 (158.96) N = 7	172.67 (112.65) N = 7	112.36 (56.33) N = 3
3403 **ZN-Α-GLYCOPROTEIN**	1.33	—	2256.80 (2077.79) N = 10	1303.60 (488.31) N = 3	3857.20 (3096.16) N = 8	3244.01 (3126.57) N = 7	4959.75 (90.43) N = 2
2902 **IG a**	1.30	1824.80 N = 1	1636.72 (1459.85) N = 4	2345.95 (1055.63) N = 2	3669.51 (2084.45) N = 9	3087.30 (2302.38) N = 10	1464.10 (1175.82) N = 7
1101 **PROLACTIN INDUCIBLE PROTEIN**	1.28	221.85 (169.21) N = 2	345.68 (262.87) N = 10	5670.8 (2673.64) N = 10	6917.96 (4527.83) N = 10	7782.34 (3910.71) N = 10	1943.79 (982.69) N = 10
3601 **ALPHA 1-ANTITRYPSIN**	1.28	356.90 (3.32) N = 2	463.42 (262.94) N = 7	1316.47 (1119.60) N = 4	343.12 (143.63) N = 8	485.22 (107.36) N = 4	642.15 (418.15) N = 8
2710 **αLPHA1-ANTITRYPSIN**	1.27	243.8 N = 1	72.5 N = 1	—	398.8 (396.30) N = 7	204.52 (195.99) N = 5	306.92 (280.82) N = 4
5501 **ZN-Α-GLYCOPROTEIN**	1.26	553.90 N = 1	1046.41 (943.96) N = 10	291.05 (36.13) N = 2	522.02 (289.49) N = 7	496.98 (344.95) N = 7	1019.70 N = 1
2702 **αLPHA1-ANTITRYPSIN**	1.25	124.6 N = 1	241.83 (132.50) N = 3	97.8 N = 1	185.08 (152.40) N = 5	77.13 (52.32) N = 6	331.8 (329.86) N = 3
6204 **IG LIGHT CHAIN**	1.25	754.6 N = 1	752.64 (1083.45) N = 5	817.45 (624.99) N = 6	238.9 (103.36) N = 5	695.81 (351.43) N = 8	386.58 (99.77) N = 5
2002 **CYSTATIN C**	1.24	—	—	149.96 (62.49) N = 3	222.65 (116.75) N = 4	289.35 (174.20) N = 4	861.45 (652.36) N = 9
1006 **UN IDENTIFIED**	1.23	—	82.38 (120.03) N = 8	833.4 (825.61) N = 2	284.35 (177.13) N = 2	793.5 N = 1	752.95 (754.69) N = 2
2904 **IG A**	1.23	1492.8 (1547.33) N = 3	1487.94 (839.74) N = 5	3465.5 (2271.26) N = 10	2618.98 (1956.63) N = 9	2233.91 (1461.74) N = 9	1781.01 (814.97) N = 8
8507 **UN IDENTIFIED**	1.21	—	233.75 (13.22) N = 2	448.91 (604.82) N = 8	548.2 (542.06) N = 7	418.05 (350.38) N = 4	604.28 (296.05) N = 5
6505 **UN IDENTIFIED**	1.21	3556.8 N = 1	2488.31 (1587.53) N = 8	838.22 (641.70) N = 5	1480.3 (806.11) N = 7	2175.7 (2191.21) N = 7	1984.46 (1621.61) N = 6
4003 **UN IDENTIFIED**	1.21	—	—	208.42 (249.95) N = 7	295.38 (417.87) N = 8	316.33 (374.77) N = 3	220.6 (133.36) N = 4
7702 **UN IDENTIFIED**	1.20	4148.86 (3268.70) N = 3	3338.56 (3098.76) N = 6	1581.8 (1693.81) N = 7	1089.54 (808.98) N = 9	1534.88 (1248.49) N = 9	2125.014 (1288.67) N = 7
1507 **ZN-Α-GLYCOPROTEIN**	1.20	1279.5 N = 1	1587.31 (1178.44) N = 6	4356.77 (2420.91) N = 10	3658.72 (3223.08) N = 8	5160.97 (3608.14) N = 10	2439.26 (2376.96) N = 9
1001 **UN IDENTIFIED**	1.20	—	—	736.5 (531.96) N = 3	1156.57 (915.71) N = 10	1391.15 (1186.59) N = 2	946.48 (682.96) N = 9

Proteins with a variable of importance (VIP) above 1.2 in the orthogonal partial least squares discriminant analysis model are listed in this table. The values are reported as mean (SD) of the optical density of the protein spots.

N = number of subjects in each group where the proteins were detected, VIP = variable of importance.
